# Are They Thinking Differently? The Perceptions and Differences in Medical Disputes between Veterinarians and Clients

**DOI:** 10.3390/vetsci10050367

**Published:** 2023-05-22

**Authors:** Zih-Fang Chen, Yi-Hsin Elsa Hsu, Jih-Jong Lee, Chung-Hsi Chou

**Affiliations:** 1 Zoonoses Research Center, School of Veterinary Medicine, National Taiwan University, Taipei 10617, Taiwan; 2 Executive Master Program of Business Administration in Biotechnology, Taipei Medical University, Taipei 11031, Taiwan; 3 School of Healthcare Administration, Taipei Medical University, Taipei 11031, Taiwan; 4 Institute of Veterinary Clinical Science, School of Veterinary Medicine, National Taiwan University, Taipei 10617, Taiwan

**Keywords:** medical dispute, risk factor for medical disputes, questionnaire, veterinarians, clients, pet owner, veterinary education, communication, complaint management, medical skills, medical expense, attitudes of stakeholders

## Abstract

**Simple Summary:**

This study explored the perceptions of veterinarians and clients regarding risk factors and potential solutions for medical disputes in veterinary practices. A total of 245 respondents in Taiwan, including 125 veterinarians and 120 clients, completed an electronic questionnaire in 2022. The questionnaire covered six dimensions: medical skills, complaint management, the attitudes of stakeholders during interactions, medical expenses, clients’ perspectives, and communication modes. The results showed significant differences in the perceptions of inducing medical dispute risk factors between clients and veterinarians in veterinary practice. Both young veterinarians and clients perceived medical skills as the highest risk factor for inducing medical disputes, while experienced veterinarians disagreed. Veterinarians with medical dispute experience identified stakeholders’ attitudes during interactions as the top contributing factor. The possible solutions for veterinarians included providing clients with cost estimates and fostering empathy and compassion for clients. In contrast, clients emphasized being informed consent of treatment and expenses. They suggested that their veterinarians should provide detailed written information. The study emphasizes the importance of understanding stakeholders’ perceptions to reduce medical disputes and highlights the need for enhanced communication by young veterinarians. The findings provide valuable insights for veterinarians and clients into preventing and managing medical disputes in veterinary practices.

**Abstract:**

Medical disputes in veterinary practices are widespread; yet, a limited amount of research has been conducted to investigate the factors contributing to medical disputes. This study examined veterinarians’ and clients’ perceptions regarding risk factors and possible solutions to medical disputes. A total of 245 respondents from Taiwan, including 125 veterinarians and 120 clients, completed an electronic self-administered, semi-structured questionnaire in 2022. The questionnaire covered six dimensions: medical skills, complaint management, the attitudes of stakeholders during interactions, medical expenses, clients’ perspectives, and communication modes. The results highlighted significant differences in the perceptions of risk factors for inducing medical disputes and possible solutions between clients and veterinarians in veterinary practice. First, young veterinarians and clients perceived medical skills as the highest risk factor for inducing medical disputes, while experienced veterinarians disagreed (*p* < 0.001). In addition, veterinarians with medical dispute experience identified stakeholders’ attitudes during interactions as the top contributing factor. Second, regarding possible solutions, all veterinarians preferred offering clients cost estimates and cultivating empathy and compassion towards them. On the other hand, clients underscored the importance of obtaining informed consent for treatments and expenses and suggested that veterinarians should supply comprehensive written information to facilitate this process. This study underlies the importance of understanding stakeholders’ perceptions to mitigate medical disputes and advocates for improved communication education and training for young veterinarians. These findings provide valuable insights for veterinarians and clients, contributing to preventing and managing medical disputes in veterinary practices.

## 1. Introduction

In modern society, veterinarians encounter heightened risks of malpractice claims and medical disputes, primarily driven by deepening client–animal bonds [1] and an increased awareness of legal rights [2,3]. Perceived or real malpractice can result in medical disputes where the unsatisfactory outcomes of improper treatment may lead to litigation against veterinarians [2,4,5]. These disputes can profoundly affect veterinarians, heightening their concern about client complaints and litigation fears. For example, a 2021 survey by the British Veterinary Association revealed that 57% of veterinary staff felt intimidated by their clients’ behavior in the previous year, marking a 10% increase from 2019 [6]. This intimidation contributed to adverse mental stress, strained relationships with future clients, increased work pressure, and even career burnout [7,8].

Intriguingly, the pivotal factor influencing a client’s decision to instigate a malpractice claim or medical disagreement is not necessarily anchored to the caliber of veterinary healthcare provided. Rather, it is frequently caused by communication failures that act as the crucial catalyst [4,6,9,10,11,12]. These disputes can be induced by client dissatisfaction with the care process or clients’ inability to accept unexpected prognoses. For example, clients anticipate veterinarians to provide accurate diagnoses and compassionately explain their patients’ conditions [4,11,13]. Additionally, complaints can precipitate medical disputes when clients feel neglected or receive insufficient explanations regarding medical expenses [14,15,16,17,18].

A considerable body of research underscores the increased work stress experienced by veterinary professionals, primarily due to suboptimal communication [7,14,19,20]. Veterinarians frequently express discontent or agitation from medical disputes due to communication breakdowns with clients. These negative experiences can exacerbate physical and mental health issues, emotional fatigue, and professional burnout, leading to suicide [14,19,20,21,22]. Some studies have assessed the impact of complaints on veterinarians or support staff [14,19,23], while others have explored the underlying causes of grievances in veterinary practice. For instance, the thematic analysis of numerous veterinary medical disputes initially attributed to technical failures revealed that they mainly resulted from professional conduct deficiencies [9]. The key concerns included shortcomings in stakeholders’ attitudes (trustworthiness and honesty), medical skills (high-quality care), effective client communication (appropriate methods and complaint management), and equitable medical expenses charged to clients [10,14,24].

Conversely, effective client communication can mitigate dissatisfaction and enhance satisfaction. Prior research has demonstrated that fulfilling clients’ expectations and cultivating a positive rapport offer considerable benefits [4,25,26,27]. Notably, clients are less inclined to file formal complaints or initiate malpractice litigation against veterinarians with whom they have developed amicable relationships [11]. The most efficient approach to managing unfavorable veterinary outcomes includes minimizing the associated risks, proactively addressing clients’ frustrations to prevent escalation, and devising practical methods to identify and rectify pre-existing disappointments. Consequently, veterinarians have advocated enhancing their abilities to meet clients’ expectations [4,11,28,29,30]. Although it has traditionally been undervalued, the significance of communication and interpersonal skills is increasingly acknowledged in veterinary medicine, which may better equip veterinarians to confront anticipated challenges [6,7,24,31,32].

Nonetheless, skillful communication necessitates a thorough understanding of the expectations of the veterinarian and the clients. To date, a limited number of studies have attempted to contrast the perspectives of veterinarians and clients regarding communication and the factors contributing to medical disputes [4,7,24,33,34]. Consequently, it is crucial to devote more research efforts to enhance our comprehension. This study, therefore, examines the factors contributing to medical disputes from the differing viewpoints of veterinarians and clients. It compares their perceptions to pinpoint disparities that warrant remedial actions to prevent medical disputes and their associated risks. This research aims to understand better clients’ and veterinarians’ perceptions of selected interactions in veterinary practice. The outcome should promote improved veterinarian–client relationships and decrease dissatisfaction and disappointment, thus reducing medical dispute risks and work stress.

## 2. Materials and Methods

### 2.1. Study Design and Questionnaire

This study was conducted in November of 2022 and investigated the perceptions of inducing medical dispute risk factors and reducing medical dispute possible solutions between veterinarians and clients in Taiwan. The study adopted the validity- and reliability-tested, semi-structured questionnaire developed by Hsu (2014) [33], which was modified using a literature review, and then by focus groups of experts, professors, and experienced veterinarians. Furthermore, this study shares thematic continuity with the research published by Chen et al. [24]. Consequently, elements of their questionnaire have been incorporated, serving as a portion of the questionnaire content in the current investigation.

There are three parts to the questionnaire: the first part was about demographic characteristics, including age, gender, stakeholders (veterinarian or client), the experience of medical disputes, and the outcomes of medical disputes. The second part focused on assessing the perceptions of risk factors for inducing medical disputes, comprising twenty-three risk factor questions that belong to six dimensions [24]: medical skills, modes of communication, the attitudes of stakeholders during interactions, medical expenses, complaints management, and clients’ perspectives (see Table 1). The third part was about the measures of perceptions of possible solutions to reduce medical dispute risks, including fifteen solutions and one open-ended question.

In the questionnaire’s subsequent segment, respondents were solicited to assess, based on their prior experiences, their perceptions of each risk factor that potentially instigates a medical dispute. This evaluation employed a Likert scale of 5 points spanning from 1 to 5: “Very unlikely, unlikely, neutral, likely, very likely”, respectively. In the last part, they were asked to evaluate their perceptions of the likelihood that each possible solution reduces the medical dispute risk based on their prior experiences using a Likert scale of five points spanning from 1 to 5: “Very unlikely, unlikely, neutral, likely, very likely”, respectively. The Likert scale represents a prevalently utilized instrument for the quantification of phenomena within the realm of medical science [35]. Previous studies have employed various analytical approaches for Likert scales [36,37,38]. The assessment derived from Likert scales can be analyzed as either ordinal or interval data [36,37,38], a feature that leads to differing opinions among researchers. Certain scholars advocate for the mean as a superior measure of central tendency, rather than the median [38,39,40,41]; yet, others propose a contrary viewpoint [36,37]. This investigation received approval from the Research Ethics Committee of National Taiwan University (NTU-REC No.: 202209HS024).

### 2.2. Respondents

Within the scope of this study, two principal stakeholder groups were the subjects of surveying: veterinarians and clients. An online Google Form platform was used to disseminate the anonymous, self-executed, semi-structured questionnaire. The distribution of this questionnaire was facilitated via multiple channels, encompassing academic institutions, professional associations, and various social media platforms, such as Facebook and LINE. Veterinarians were invited to participate through email or social networks connected to veterinary medical associations and animal hospitals. 

The questionnaire was distributed through social media, such as Facebook groups and Line groups. The responses from veterinarians and clients were anonymously collected. Before responding, respondents were presented with an informed consent statement outlining the survey details, including estimated completion time and confidentiality measures. Respondents proceeded to complete the questionnaire upon giving informed consent. Due to the anonymous nature of the online Google Form, the response rate is not reported.

### 2.3. Analysis

Demographic attributes, including age, gender, experiences of medical disputes, and the outcomes of these disputes, were synthesized by employing descriptive statistical methodologies. These encompassed quantitative measures such as counts, averages, and percentages. The perceptions of potential risk factors leading to medical disputes and possible solutions to reduce such disputes were quantified across six dimensions using a five-point Likert scale. These responses were treated as continuous variables and subjected to statistical analysis, specifically determining their means and standard deviations.. The t-test was utilized to compare the perceptions of inducing medical dispute risk factors between stakeholders, genders, and experiences of medical disputes. t-test statistical analyses were executed using SPSS statistical software, maintaining a significance level of *p* < 0.05. In the questionnaire, we added an open-ended question for collecting veterinarians’ and clients’ perspectives of approaches to reducing medical risks. The gathered responses were carefully organized, categorized, and investigated to reveal underlying implications.

## 3. Results

A total of 253 respondents took part in the survey, consisting of 125 veterinarians and 128 clients. However, the analysis ultimately excluded eight respondents due to several factors, including being below 20 years of age, finishing the survey in an implausibly short time (less than 1 min), or providing an excessive number of incomplete responses. As a result, the final analysis included data from 125 veterinarians and 120 clients.

### 3.1. Demographic Characteristics

A total of 245 individuals participated in the questionnaire, which included 125 veterinarians and 120 clients. In terms of age distribution, both veterinarians and clients were primarily concentrated in the 30–39 and 40–49 age groups (see Table 2). These groups accounted for 71.2% of veterinarians and 71.66% of clients. Only 8 veterinarians (6.4%) were over 50, while 14 clients (11.7%) were in this age group. Regarding gender distribution (see Table 2), most veterinarians and clients were female, with 56% of veterinarians and 95.8% of clients being women. Male clients only made up 4.2%. In the experiences of medical disputes category (see Table 2), a high percentage (90.4%) of veterinarians had experienced medical disputes. In contrast, only 33 clients (27.5%) reported having these experiences. When examining the outcomes of medical disputes experienced by respondents (see Table 2), it was found that nearly 50 veterinarians (40%) resolved disputes through simple communication. An additional 53 veterinarians (42.4%) resolved disputes through communication combined with financial compensation or reduced medical fees, totaling 82.4%. Of clients, 14 were unclear about the outcomes, while roughly 60% of disputes were resolved through simple communication or financial compensation/reduced fees. Concerning third-party intervention in dispute resolution, 22 veterinarians (17.6%) and 34 clients (28.3%) endeavored to resolve conflicts utilizing this approach. In most instances involving third-party participation, approximately sixteen veterinarians and eight clients reached agreements through mediation and financial compensation. In addition, one veterinarian and two clients settled medical disputes through third-party intervention and straightforward communication. Out of the 125 veterinarians surveyed, only 4% resorted to legal proceedings. Conversely, the clients demonstrated a higher propensity (20%) to pursue legal recourse even after engaging in third-party mediation.

Veterinarians with medical dispute experience had an average age of 36.88 (SD = 8.31). In contrast, those without dispute experience had an average age of 31.50 years (SD = 7.99), which represents a statistically significant difference (*p* = 0.03). For clients, those with medical dispute experience had an average age of 37.76 years (SD = 8.63), while those without dispute experience had an average age of 38.98 years (SD = 9.28); however, no statistically significant difference was observed (see Table 3).

### 3.2. Ranking of the Perceptions of Risk Factors for Inducing Medical Disputes in Six Dimensions

This study evaluated the respondents’ perceptions of inducing medical dispute risk factors using a five-point Likert scale. The subsequent analysis involved the calculation of mean and standard deviation. Higher scores indicated a greater likelihood of a specific risk factor contributing to medical disputes. The six dimensions of risk factors for inducing medical disputes were arranged in descending order based on their mean scores (refer to Table 4). For example, veterinarians with medical dispute experience identified the attitudes of stakeholders during interactions, complaint management, and medical expenses as the top three contributing factors to the risk of medical disputes. In contrast, clients considered medical skills, complaints management, and medical expenses as the top three factors.

Meaningful comparisons were observed between the top risk factors for inducing medical disputes recognized by veterinarians and clients. Veterinarians regarded attitudes during interactions between veterinarians and clients as the primary risk factor leading to medical disputes, while clients identified medical skills as the main factor (see Table 4). Interestingly, younger veterinarians without medical dispute experience exhibited views similar to those of clients concerning the risk levels associated with factors contributing to medical disputes (see Table 4).

### 3.3. Differences in Perception of Risk Factors for Inducing Medical Disputes between Veterinarians and Clients

Veterinarians and clients demonstrated statistically significant differences in the perceptions of four risk factor dimensions induced by medical disputes (see Table 5). For instance, clients assigned noticeably higher scores in specific dimensions of inducing medical dispute risk factors, such as medical skills (clients: 4.64 ± 0.55; veterinarians: 4.13 ± 0.84, *p* < 0.001) and complaints management (clients: 4.45 ± 0.61; veterinarians: 4.20 ± 0.60, *p* = 0.002). Conversely, veterinarians perceived the risk level of stakeholders’ attitudes during interactions leading to medical disputes to be significantly higher than that of clients (veterinarians: 4.26 ± 0.57, clients: 4.09 ± 0.70, *p* = 0.038), and also, believed that clients’ perspectives posed a significantly higher risk of medical disputes than the clients themselves acknowledged (veterinarians: 4.07 ± 0.65, clients: 3.88 ± 0.67, *p* = 0.04) (see Table 5).

Upon further analysis, by comparing perspectives based on medical dispute experience, both veterinarians and clients exhibited similar perceptions of the risk factor dimensions leading to medical disputes (see Table 5), particularly in the areas of medical skills (clients: 4.71 ± 0.48; veterinarians: 4.12 ± 0.85, *p* < 0.001) and complaints management (clients: 4.60 ± 0.53; veterinarians: 4.19 ± 0.6, *p* < 0.001). Clients assigned significantly higher scores in these two dimensions seemed to agree on the risk level associated with these factors. Although veterinarians’ scores for complaints management appeared lower than those of the clients, experienced veterinarians ranked it as the second-highest risk factor for medical disputes, suggesting that they also placed considerable importance on this aspect (see Table 5). However, the medical dispute risk factor of medical skills was ranked fourth by veterinarians. Despite this, the dimension’s score was still above four (likely to induce medical disputes), indicating that veterinarians recognized its importance, but held a different view than that of the clients regarding ranking and evaluation (see Table 5).

### 3.4. The Perceptions of Veterinarians and Clients on Possible Solutions to Reduce the Medical Disputes Risk

In the third section of the questionnaire, utilizing a five-point Likert scale, this study assessed the perceptions of veterinarians and clients of fifteen potential solutions for reducing the risk of medical disputes (see Table 6). Table 6 lists the potential solutions and the dimensions they belong to and the abbreviations of the solutions. In addition, the fifteen possible solutions for reducing medical disputes could be grouped into six dimensions: medical skills, modes of communication, the attitudes of stakeholders during interactions, medical expenses, complaints management, and training and education.

The subsequent analysis involved calculating the mean and standard deviation, with higher scores signifying a greater likelihood of a solution effectively reducing the medical dispute risks evaluated by veterinarians and clients. The top five solutions were then arranged in descending order based on their mean scores for veterinarians and clients (see Table 7).

For instance, veterinarians regarded “Providing clients with a possible cost range estimate before starting treatment and explaining it clearly (Cost_Esti)” as the most effective solution (see Table 7), followed by “Encouraging veterinarians to express empathy and compassion during interactions with clients (Emp_Compa)” and “Urging clients to articulate their concerns and questions clearly, allowing veterinarians to address them promptly and thereby increasing trust (Ques_Expres)”. Additionally, veterinarians with medical dispute experience ranked “Informing clients of potential risks and treatment outcomes related to patients’ diseases, enabling them to make informed decisions (Info_OC)” as the second most effective solution. However, different from the viewpoints of veterinarians, clients identified “Explaining risks and having clients sign a consent form before performing surgical procedures or treating critically ill patients (Cons_Fo)” as the most effective solution (see Table 7).

The study also included one open-ended question to gather other potential solutions. Thirty-two respondents (13%) provided valuable insights, with sixteen veterinarians and sixteen clients, summarized as three key points:

Point 1: The importance of communication courses and training. As mentioned by ten veterinarians and ten clients, the main suggestions were as follows: “Offer communication skills, empathy-related courses, or psychology-related courses to hospital staff, veterinarians, and future veterinary students to develop empathy and communication skills” (38-year-old, female client), “Enhance the cooperative relationship between medical personnel and clients through regular training and health education, fostering mutual trust and achieving a win-win situation” (36-year-old, female client), and “Emphasize the importance of empathy among veterinarians and staff, as pets are considered family members by their owners” (56-year-old, female client).

Point 2: Having enough time and information for communication and decision making. As mentioned by five veterinarians and seven clients, the main suggestions were as follows: “Implement an appointment system to provide sufficient communication time for both parties” (36-year-old, male veterinarian) and “Involve clients in medical decision-making (Shared Decision Making) to encourage informed decision-making and shared responsibility” (43-year-old, male veterinarian).

Point 3: Surveillance video systems. As mentioned by two veterinarians and one clients, the main suggestion was as follows: “Install surveillance video systems in clinics to document treatment procedures.” (51-year-old, female veterinarian).

These suggestions were condensed into three possible solutions: “Time Management and Appointment Control”, “Install Surveillance Systems in Clinics to Document Treatment Procedures”, and “Involve Clients in Decision-Making (Shared Decision Making)”. We summarized these potential solutions recommendations with valuable insights into critical facets/items according to six dimensions and presented them in Table 8.

## 4. Discussion

The present study examined the perceptions of risk factors and possible solutions for medical disputes between veterinarians and clients, revealing several noteworthy findings. The results noted shared perspectives between the two groups on certain elements, yet there were marked differences in their perceptions regarding the risk factors that could instigate medical disputes and potential solutions to alleviate them.

### 4.1. Age and Medical Dispute Experience

One interesting observation was the significant difference in age between veterinarians with and without medical dispute experience. This observation might be explained by the fact that older veterinarians are more likely to have encountered many more cases. Therefore, the risk of conflicts is much higher due to the number of cases handled.

Previous research has indicated that older physicians may possess more clinical experience. Those with more years of practice may encounter more medical disputes due to their exposure to more cases [29,42]. According to the findings by Hickson et al., there was no significant difference in the risk of complaints between physicians aged 26–35 and those aged ≤25. However, older practitioners faced a 1.5-to-2.1-times-higher risk of receiving complaints, which generally increases with age [29]. Nevertheless, veterinarians with more years of practice may have more medical skills experience, enabling them to identify risks of complaints earlier and address them promptly, potentially reducing their likelihood of encountering medical disputes [18,28,43]. 

Fortunately, to mitigate the risk of medical disputes, relying solely on age or years of practice to accumulate skills and experience handling these issues is unnecessary. Through practical training and sharing case experiences, understanding clients’ needs and expectations can effectively reduce complaints and medical disputes. Veterinarians with continuous education can significantly improve their abilities in preventing disputes and managing complaints [11,22,31,32,43].

### 4.2. Perceptions of Risk Factors for Inducing Medical Dispute Risks

Veterinarians and clients exhibited differing perceptions of risk factors for inducing medical disputes. Veterinarians emphasized the attitudes of stakeholders during interactions, while the clients were more concerned about medical skills. Intriguingly, younger veterinarians without medical dispute experience shared similar perspectives to those of clients regarding the risk levels associated with factors contributing to medical disputes. A cross-sectional study conducted between 2014 and 2022 found that young veterinarians and veterinary students with no dispute experience often perceive medical skills as the primary risk factor for medical disputes [24]. This divergence in perspectives might stem from experienced veterinarians’ professional knowledge and experience, making them consider the importance of communication in maintaining positive relationships with clients [2,6,23]. Moreover, a 1995 study in Taiwan by Yang et al. investigated the perspectives of 99 clients to determine the criteria for a “good veterinarian”. The main factors identified were “accurate diagnosis” and “detailed explanation”. These findings imply that clients generally lack medical expertise and might prioritize veterinarians’ technical competence in treating their pet patients [34]. Interestingly, these results coincide with the findings of the current study. However, numerous studies highlight that although a lack of medical expertise contributes to the risk of medical disputes, ineffective communication with clients represents the most significant factor. Poor communication can undermine trust and hinder establishing a robust collaborative relationship, ultimately leading to clients’ complaints and potential medical disputes [6,9,10,14,22,23].

### 4.3. Perceptions of Possible Solutions to Reduce Medical Dispute Risks

Both veterinarians and clients identified potential solutions for reducing medical dispute risks, with similarities and differences in their preferences. For instance, providing cost estimates and fostering empathy and compassion were highly valued by veterinarians, while obtaining informed consent and providing written information were considered to be crucial by clients [23,34].

Studies concerning reducing the number of client complaints and medical dispute risks have emphasized explaining medical costs. Communication skills and training methods in human medicine and veterinary medicine are similar, as both aim to improve the interactions between healthcare providers and professionals dedicated to enhancing patients’ health [30].

Empathy, a fundamental communication skill, is particularly relevant when one is discussing medical costs. Providing detailed explanations of costs ensures that clients understand the expenses and prognosis, allowing them to accept the proposed treatment’s financial implications [10,17,18,23,30]. Moreover, research on increasing client satisfaction indicates that when veterinarians establish trust with clients, client satisfaction improves, as does job satisfaction and the sense of accomplishment for veterinarians [44].

The open-ended responses provided additional insights into potential strategies for reducing medical disputes, such as time management and appointment control, installing surveillance systems in clinics [2,9], and involving clients in decision making [6,23,45]. However, research has shown that the duration of each visit may not necessarily be related to client satisfaction with the veterinarian. Instead, open-ended questions during communication allow the clients to express their concerns and doubts [4,6,23]. In addition, effective communication and time management in appointment systems can be achieved through the expertise and experience of veterinarians [44]. Lastly, veterinary-related research on shared decision making often stems from the significant knowledge gap between veterinarians and clients. Veterinarians must provide relevant knowledge and specific advice, express support, and enable clients to make informed decisions [6,45].

These preferences may reflect each group’s priorities and concerns, with veterinarians being focusing on clear communication and compassionate care, and clients emphasizing transparency and shared decision making in their pets’ treatment. These suggestions underscore the importance of improving communication between veterinarians and clients, enhancing the transparency of treatment procedures and fostering a collaborative approach to healthcare.

### 4.4. Limitations and Implications for Future Research

The findings of this study have important implications for veterinary practice, as they emphasize the need for effective communication, empathy, and shared decision making in the prevention and resolution of medical disputes. Veterinary professionals should be aware of these factors and consider implementing strategies to address them, such as providing cost estimates, obtaining informed consent, and offering written information to clients.

Despite its valuable insights, the study bears several constraints that warrant consideration. Initially, the investigation was conducted in Taiwan, characterized by a comparatively diminutive veterinarian population relative to those of other nations. Consequently, the applicability of the findings to countries with divergent healthcare frameworks and cultural contexts might be limited. Secondly, the study hinged on self-reported data, potentially rendering it susceptible to social desirability bias. This might have led participants to furnish responses that are perceived to be socially acceptable, rather than those that genuinely mirror their authentic perceptions. Alternatively, exploring human psycho-social and emotional contexts presents considerable challenges. Nonetheless, undertaking comparative analyses in these domains could be of significant importance. The same sentiment could not be expected from clients who lost their companion animals and someone whose animals only spent a long time hospitalized due to a medical error. Gaining insights into these factors could provide a valuable understanding of the reasons behind evolving perceptions.

As a result, this method precluded the extraction of more profound insights from the study. Ultimately, despite the frequent use of the Likert scale to obtain interval data within medical research, it is intrinsically ordinal. Consequently, the data were analyzed according to the median rather than the mean, a factor that could potentially impact the interpretation of the findings.

Despite these constraints, the study offers invaluable perspectives into the understanding of risk factors instigating medical disputes between veterinarians and clients. Future investigations may delve into the elements contributing to the risk perception among diverse stakeholders, compare the perception of risk factors inciting medical disputes across different countries, and scrutinize the efficacy of potential solutions aimed at mitigating the risk of medical disputes. Furthermore, a future study could be designed that selects participants who have previously handled or were involved in medical disputes and excludes non-experienced participants. Future investigations could additionally explore the effectiveness of various continuing education programs in cultivating practical communication competencies and complaint management techniques among emerging veterinarians. In conclusion, an understanding of clients’ perceptions can foster enhanced relationships and elevate the quality of care provided to companion animals, diminish the likelihood of medical disputes, and improve the expertise of veterinary practitioners.

In conclusion, this study offers valuable insights into the perceptions of risk factors and possible solutions for medical disputes between veterinarians and clients. The findings highlight the importance of communication, empathy, and shared decision making in preventing and resolving medical disputes and suggest potential avenues for further research and practical interventions to enhance the quality of care in veterinary practice. Collaborative efforts between veterinarians and clients can contribute to more effective communication, greater transparency, and improved client satisfaction, ultimately leading to decreased medical disputes and a better overall experience for both parties. By fostering a strong partnership between veterinarians and clients, it is possible to achieve high-quality care and better outcomes for companion animals.

## Figures and Tables

**Table 1:** Six questionnaire dimensions for measures of perceptions of risk factors for inducing medical disputes.

Dimensions	Risk Factors That Might Induce Medical Disputes
Medical skills	Misdiagnosis that results in the deterioration of the patient’s condition. Inadequate or unsuitable hospital care or treatment protocols.
Modes of communication	The elucidations offered by veterinarians to clients are overly simplified. The use of excessive medical terminology during explanations without providing comprehensive clarifications. Clients harbor uncertainties about the treatment yet either neglect to seek clarification or receive unsatisfactory responses from the veterinarians before the treatment. A disparity between clients’ anticipated and actual treatment outcomes, attributable to an information deficit impeding decision-making before treatment. The absence of decisive action from the clients’ families during discussions about further treatment procedures with the veterinarian. The veterinarians do not furnish additional written resources to clients to enhance their understanding of the treatment.
The attitudes of stakeholders during interactions	Veterinarians endeavor to induce clients into reluctant acceptance of the proposed diagnosis and treatments. Veterinarians fail to respond suitably or implement effective measures to address clients’ concerns. Veterinarians do not convey feelings of support and encouragement during client interactions, thereby leading to the perception that they may be indifferent to the wellbeing of the afflicted animals. In instances where the perspectives of clients and veterinarians diverge on the animal’s condition, the client tends to adhere to their subjective perception.
Medical expenses	Veterinarians fail to communicate potential comprehensive medical expenses to clients beforehand. Veterinarians do not explicitly elucidate the possible medical costs of each treatment procedure. The divergence between clients’ anticipated and actual medical expenditures.
Complaints management	Veterinarians exhibit no initiative in promptly addressing the complaints. There is an absence of senior personnel attending to the complaints. The complaint resolution process is conducted with inappropriate attitudes and improper methods. Staff members lack the relevant professional training necessary for managing the complaints.
Client’s perceptions	Clients rely on self-acquired information. Clients possess numerous inquiries regarding treatment and manifest a deficiency of trust in the interaction. Clients harbor misconceptions about their pets being attended to by the veterinarian. Clients might potentially harbor ulterior motives of extortion.

**Table 2:** Demographic characteristics distribution of veterinarians and clients.

Characteristics	Veterinarians (*n* = 125)		Clients (*n* = 120)	
	N	%	N	%
Age				
20–29 years old	28	22.40%	20	16.67%
30–39 years old	55	44.00%	46	38.33%
40–49 years old	34	27.20%	40	33.33%
50–years old	8	6.40%	14	11.67%
Gender				
Male	55	44.00%	5	4.17%
Female	70	56.00%	115	95.83%
Experiences of medical disputes				
Experienced	113	90.40%	33	27.50%
Did not experience	12	9.60%	87	72.50%
Outcomes of medical disputes				
No idea	0	0.00%	14	11.67%
Resolved by simple communication.	50	40.00%	37	30.83%
Resolved by communication and reconciliation via money compensation or medical fee reduction.	53	42.40%	35	29.17%
Resolved/unresolved by third-party involvement/mediation.	22	17.60%	34	28.34%
-Resolved by third-party involvement/mediation and simple communication.	1	0.80%	2	1.67%
-Resolved by third-party involvement/mediation and reconciliation via money compensation or medical fee reduction.	16	12.80%	8	6.67%
-Unresolved even with third-party involvement and the complaint was filed in the court.	5	4.00%	24	20.00%

**Table 3:** Comparison of age differences between the subgroups of the stakeholder with and without medical dispute experience: the cases of veterinarians and clients.

Subgroups	Veterinarians’ Age				Clients’ Age			
	N (%)	Mean	SD	*p*	N (%)	Mean	SD	*p*
Experiences of medical disputes	113 (90.4%)	36.88	8.31	0.03 *	33 (27.5%)	37.76	8.67	0.05
Without experiences of medical disputes	12 (9.6%)	31.5	7.99		87 (72.5%)	38.98	9.28	

*p* value < 0.05 *.

**Table 4:** Ranking of the perceptions of risk factors for inducing medical disputes in six dimensions for veterinarians and clients.

Dimensions of Risk Factors for Inducing Medical Disputes	Veterinarians						Clients					
	All		With Experiences of Medical Disputes				All		With Experiences of Medical Disputes			
	*n* = 125		Yes (*n* = 113)		No (*n* = 12)		*n* = 120		Yes (*n* = 33)		No (*n* = 87)	
	Mean	Rank ^1^	Mean	Rank	Mean	Rank	Mean	Rank	Mean	Rank	Mean	Rank
Attitudes of stakeholders during interactions	4.26	1	4.27	1	4.23	4	4.09	4	4.12	4	4.08	3
Complaints management	4.20	2	4.19	2	4.25	3	4.45	2	4.6	2	4.39	2
Medical expenses	4.18	3	4.17	3	4.28	2	4.10	3	4.23	3	4.05	4
Medical skills	4.13	4	4.12	4	4.29	1	4.64	1	4.71	1	4.61	1
Clients’ perspectives	4.07	5	4.06	5	4.17	5	3.88	6	3.89	6	3.88	6
Modes of communication	3.98	6	3.98	6	4.01	6	3.99	5	3.98	5	3.99	5

^1^ Rank by the mean from highest to lowest.

**Table 5:** Differences in perception of risk factors for inducing medical disputes between veterinarians and clients by experiences of medical disputes.

Dimensions of Risk Factors for Inducing Medical Disputes	All				*p*	With Experiences of Medical Disputes				*p*
	Veterinarians n = 125		Clients n = 120			Veterinarians n = 113		Clients n = 33		
	Mean	SD	Mean	SD		Mean	SD	Mean	SD	
Attitudes of stakeholders during interactions	4.26	0.57	4.09	0.70	0.038 *	4.27	0.59	4.12	0.68	0.229
Complaints management	4.20	0.6	4.45	0.61	0.002 **	4.19	0.6	4.6	0.53	0.001 **
Medical expenses	4.18	0.67	4.10	0.83	0.414	4.17	0.68	4.23	0.81	0.648
Medical skills	4.13	0.84	4.64	0.55	<0.001 ***	4.12	0.85	4.71	0.48	<0.001 ***
Clients’ perspectives	4.07	0.65	3.88	0.76	0.04 *	4.06	0.64	3.89	0.72	0.204
Modes of communication	3.98	0.56	3.99	0.67	0.909	3.98	0.56	3.98	0.74	0.953

*p* value < 0.05 *; *p* value < 0.01 **; *p* value < 0.001 ***.

**Table 6:** The abbreviation of possible solutions for medical disputes.

Dimensions	Possible Solutions for Reducing the Medical Dispute Risks	Abbreviations
Medical skill	Encourage veterinarians to improve their medical skills through on-the-job training and seminars.	Impr_MS
	Use checklists during the diagnostic and treatment to avoid overlooking details and mistakes.	Use_CL
	To prevent errors and disputes, encourage veterinarians to use forms or records and provide clear instructions during patient handoffs.	Handoffs
Modes of communication	Design educational materials like pamphlets or brochures with disease-related information to increase clients’ understanding, reduce misunderstandings, and avoid negligence.	Des_Mate
	Inform clients of the potential risks and treatment outcomes related to patients’ diseases, enabling them to make informed decisions.	Info_OC
	Explain the risks and have clients sign a consent form before performing surgical procedures or treating critically ill patients.	Cons_Form
	Ensure mutual confirmation and agreement on subsequent treatment and provide written information to clients, even for non-surgical or non-critical incidents.	Writ_Info
Attitudes of stakeholders during interactions	Encourage veterinarians to express empathy and compassion during interactions with clients.	Emp_Compa
	Encourage clients to express their concerns and questions clearly, allowing veterinarians to address them promptly, thereby increasing trust.	Ques_Expres
Medical expenses	Provide clients with a possible cost range estimate before starting treatment and explain it clearly.	Cost_Esti
Complaints management	Offer continuing education courses for colleagues, such as clinical communication, handling customer complaints, and medical disputes.	CC_Course
	Address complaints promptly to reduce negative impacts.	Addr_CC
Education and training	Organize public education seminars by the clinic or veterinary organizations to educate clients on proper pet care practices and effective communication with veterinarians.	Edu_Client
	Organize in-house employee training to improve communication skills, such as practical discussions and case study.	In_House
	Add education courses related to medical disputes and customer complaints, such as communication skills and handling customer complaints, to the veterinary curriculum at universities.	Uni_Course

**Table 7:** Ranking of the perceptions of possible solutions for veterinarians and clients.

Rank ^1^	Veterinarians			Clients		
	All *n* = 125	With Experiences of Medical Disputes		All *n* = 120	With Experiences of Medical Disputes	
		Yes (*n* = 113)	No (*n* = 12)		Yes (*n* = 33)	No (*n* = 87)
Top 1 (Mean)	Cost_Esti (4.42)	Cost_Esti (4.40)	Cost_Esti (4.67)	Cons_Form (4.76)	Cons_Form (4.88)	Cons_Form (4.71)
Top 2 (Mean)	Emp_Compa (4.39)	Info_OC (4.39)	Emp_Compa (4.58)	Ques_Expres (4.73)	Info_OC (4.88)	Ques_Expres (4.70)
Top 3 (Mean)	Ques_Expres (4.38)	Emp_Compa (4.37)	Ques_Expres (4.42)	Info_OC (4.71)	Writ_Info (4.88)	Cost_Esti (4.68)
Top 4 (Mean)	Info_OC (4.36)	Ques_Expres (4.37)	Addr_CC (4.42)	Handoffs (4.68)	Addr_CC (4.82)	Handoffs (4.66)
Top 5 (Mean)	Addr_CC (4.31)	Addr_CC (4.30)	Writ_Info (4.42)	Addr_CC (4.67)	Ques_Expres (4.79)	Emp_Compa (4.66)

^1^ Rank by the mean from highest to lowest.

**Table 8:** Modified possible solutions to reduce the medical dispute risks.

Dimensions	Possible Solutions for Reducing the Medical Dispute Risks
Medical skill	Encourage veterinarians to improve their medical skills through on-the-job training and seminars.
	Use checklists during the diagnostic and treatment to avoid overlooking details and mistakes.
	To prevent errors and disputes, encourage veterinarians to use forms or records, and provide clear instructions during patient handoffs.
Modes of communication	Design educational materials, such as pamphlets or brochures with disease-related information, to increase clients’ understanding, reduce misunderstandings, and avoid negligence.
	Inform clients of the potential risks and treatment outcomes related to patients’ diseases, enabling them to make informed decisions.
	Explain the risks and have clients sign a consent form before performing surgical procedures or treating critically ill patients.
	Ensure mutual confirmation and agreement on subsequent treatment and provide written information to clients, even for non-surgical or non-critical incidents.
	Control appointment times and allow enough time for communication between veterinarians and clients. (Newly added,)
	Involve clients in decision-making to increase trust and collaboration (shared decision making). (Newly added.)
	Install surveillance systems in clinics to document treatment procedures for records. (Newly added.)
Attitudes of stakeholders during interactions	Encourage veterinarians to express empathy and compassion during interactions with clients.
	Encourage clients to express their concerns and questions clearly, allowing veterinarians to address them promptly, thereby increasing trust.
Medical expenses	Provide clients with a possible cost range estimate before starting treatment and explain it clearly.
Complaints management	Offer continuing education courses for colleagues, such as clinical communication, handling customer complaints, and medical disputes.
	Address complaints promptly to reduce negative impacts.
Education and training	Organize public education seminars by the clinic or veterinary organizations to educate clients on proper pet care practices and effective communication with veterinarians.
	Organize in-house employee training to improve communication skills, such as practical discussions and case study.
	Add education courses related to medical disputes and customer complaints, such as communication skills and handling customer complaints, to veterinary curriculums at universities.

## Data Availability

Not applicable.
